# Polycyclic aromatic hydrocarbon derivative 3-hydroxybenz[a]anthracene promotes the progression of T47D breast cancer cells by modulating relevant protein expression

**DOI:** 10.3389/fcell.2026.1794498

**Published:** 2026-05-20

**Authors:** Wenting Song, Xiao Kang, Xueyan Li, Yuyan Yang, Xinke Wu

**Affiliations:** 1 School of Medicine, Henan Polytechnic University, Jiaozuo, China; 2 Jiaozuo Key Laboratory for Huaiyao Comprehensive Development, Henan Polytechnic University, Jiaozuo, Henan, China

**Keywords:** 3-hydroxybenz[a]anthracene, breast cancer cells, estrogen-like activity, cell progression, protein regulation

## Abstract

Polycyclic aromatic hydrocarbon derivatives, as a class of environmental pollutants, often exhibit higher toxicity than their parent polycyclic aromatic hydrocarbons, posing potential health risks. This study selected the potentially estrogenic derivative 3-hydroxybenz[a]anthracene as the research subject. Using the estrogen receptor-positive breast cancer cell line T47D as a model, the effects of this compound on cell proliferation, migration, invasion, and apoptosis were evaluated through EdU staining, colony formation, scratch healing, Transwell invasion, and apoptosis assays to evaluate its effects on cell proliferation, migration, invasion, and apoptosis. Western blot analysis was employed to detect the expression of relevant signaling proteins. Results indicate that 3-hydroxybenz[a]anthracene promotes T47D cell proliferation by activating the PI3K/AKT signaling pathway, thereby upregulating AKT, p-AKT, and c-Myc protein expression. It enhances cell migration and invasion by downregulating E-Cadherin and MMP9 while simultaneously upregulating Vimentin and MMP2 protein expression. Furthermore, this compound simultaneously upregulates Bax and Bcl-2 expression, ultimately inducing apoptosis in T47D cells. This study confirms that 3-hydroxybenz[a]anthracene exhibits estrogen-like activity *in vitro* and can influence malignant biological behaviors of breast cancer cells by regulating relevant signaling pathways. These findings provide experimental evidence for further evaluating the endocrine-disrupting effects and breast cancer risks associated with such environmental pollutants.

## 1 Introduction

Breast cancer is ranked among the globally most prevalent cancers affecting women, having a notably high incidence rate and a clear recent trend toward younger populations (Peng et al., 2024). While breast cancer treatment options continually diversify, many therapies are associated with considerable side effects, substantial financial burden, and the risk of drug resistance and disease recurrence following long-term medication (Li et al., 2025). Therefore, alongside actively refining treatment protocols, strengthening prevention at its source is of paramount importance. The development of breast cancer is related to multiple contributing factors, such as genetic predisposition, prolonged emotional stress, living environment, and irregular lifestyle patterns (Niebauer et al., 2021). Among them, the relationship between the living environment and breast cancer development warrants particular attention since long-term exposure to polluted environments may increase the risk of several diseases, including breast cancer (Quoc et al., 2023). Despite the beneficial effects of industrialization, it has also led to varying degrees of pollution in the air, water, and soil, so actively mitigating and avoiding environmental risks should be a crucial component of breast cancer prevention strategies.

Polycyclic aromatic hydrocarbons (PAHs) and their derivatives are a class of organic compounds widely present in the environment. PAHs consist of two or more fused benzene rings (Montano et al., 2025) and exhibit high molecular weight, elevated melting and boiling points, low vapor pressure, strong hydrophobicity, poor biodegradability, and chemical stability (Zhang X. et al., 2023). The majority of PAHs are carcinogenic, teratogenic, and mutagenic (Cheng et al., 2023; Ju et al., 2022). PAHs primarily originate from human activities, e.g., barbecuing, automobile emissions, and industrial wastewater discharges (Petrón et al., 2025; Wang et al., 2022; Wu et al., 2020), which closely delineate the main PAHs exposure routes: inhalation, ingestion, and dermal contact (Zhang et al., 2025). PAHs and their derivatives were reported to increase cancer risk in humans (Lee et al., 2020), and multiple PAHs were classified as potential carcinogens and endocrine disruptors (Hwang et al., 2019). Furthermore, PAHs exposure may elevate the risk of cardiovascular diseases, including atherosclerosis, hypertension, thrombosis, and myocardial infarction (Mallah et al., 2022). The formation of PAH derivatives via oxidation, nitration, hydroxylation, and alkylation, and in complex ecological environments, is also of high interest (Zhang Y. et al., 2023) because they often exhibit stronger carcinogenic and mutagenic potential, although they typically exist at lower concentrations than their parent PAHs (Liu et al., 2020). Therefore, PAH derivatives should be further studied.

Epidemiological studies indicated that exposure to PAHs and their derivatives increased the risk of breast cancer incidence and mortality (Amadou et al., 2021; Mordukhovich et al., 2016; Shen et al., 2017), but little research was devoted to the estrogenic effects of these compounds on breast cancer. 3-hydroxybenz[a]anthracene (3-OH-B[a]A) is not only a ubiquitous atmospheric pollutant (Honda et al., 2022), but also one of the primary hydroxylated metabolites of benz[a]anthracene, the parent compound of PAHs, in human urine (Gündel and Angerer 2000). Competition assay and yeast two-hybrid assay expressing human estrogen receptor (hER) showed that 3-OH-B[a]A can bind to hER and exhibit estrogenic activity (Hayakawa et al., 2008). However, its estrogen-like effects on breast cancer cells and the underlying toxicological mechanisms were not systematically studied. Therefore, we selected the estrogen receptor-positive breast cancer T47D cell line as a model to investigate the effects of 3-OH-B[a]A on cellular proliferation, migration, invasion, and apoptotic behavior and determine the underlying molecular mechanisms. These findings provide scientific evidence for the estrogen-mimetic activity of PAH derivatives and shed light on their role in breast cancer development, laying a foundation for related risk assessment and prevention strategies.

## 2 Materials and methods

### 2.1 Materials

3-hydroxybenz[a]anthracene (3-OH-B[a]A) was purchased from Shanghai Aladdin Bio-Chem Technology Co., Ltd (Shanghai, China). 17β-estradiol (E_2_) was obtained from Shandong Sikejie Biotechnology Co., Ltd (Jinan, China). The stock solutions were prepared by dissolving the chemical in dimethylsulfoxide (DMSO, MP Biomedicals) and kept at 4 °C in darkness. Activated charcoal-treated fetal bovine serum was purchased from Beijing Zoman Biotechnology Co., Ltd (Beijing, China). One-Step TUNEL Apoptosis Detection Kit were obtained from Abbkine Scientific Co., Ltd (Wuhan, China). Cell-Light EdU Apollo567 *in vitro* kit was purchased from Guangzhou Ribo Bio Co., Ltd. (Guangzhou, China). Triton X-100 and Glycine were purchased from Macklin Corporation (Jinan, China). Crystal violet saining solution was purchased from Shanghai Beyotime Biotechnology Co., Ltd. (Shanghai, China). Paraformaldehyde was obtained from Wuhan Servicebio Technology Co., Ltd. (Wuhan, China). Matrigel matrix was purchased from ABW (Shanghai, China).

### 2.2 Cell line, culture and working solutions

Human breast cancer cell T47D was used as the *in vitro* assay model in this paper. T47D cells were cultured in 1640 (Sikejie, Jinan, China) containing 10% fetal bovine serum (FBS, Abbkine, Wuhan, China), 100 U/mL penicillin, and 100 μg/mL streptomycin (Sikejie, Jinan, China). The incubations were kept in a humidified atmosphere of 5% CO_2_ at 37 °C.

The 3-OH-B[a]A mother liquor was diluted into 0.4 μM and 2 μM working solution with complete culture medium; the E_2_ mother solution was diluted into 0.5 nM and 5 nM working solution, and the final concentration of DMSO is ≤ 0.1%. The concentrations of 3-OH-B[a]A used in this study (0.4 μM and 2 μM) were selected based on preliminary E-screen assays that showed clear dose–response relationships. In these assays, exposure to 0.4 μM and 2 μM 3-OH-B[a]A resulted in the two highest increases in MCF-7 cell viability after 48 h, with a significant increase observed at 0.4 μM (unpublished data). Effects and underlying mechanisms of 0.4 μM and 2 μM 3-OH-B[a]A on the progression of T47D cells should be further investigated.

Based on preliminary screening, 0.5 nM E_2_ was selected as the positive control for cell proliferation and apoptosis assays, and 5 nM E_2_ was selected as the positive control for cell migration and invasion assays.

### 2.3 Antibobies

Primary antibodies used were: rabbit monoclonal to β-Actin (Beyotime, Shanghai, China, Cat # AF5003; 1:1000 dilution); mouse monoclonal to AKT (Proteintech, Wuhan, China, Cat # 60203-2-Ig; 1:5000 dilution); rabbit monoclonal to p-AKT (Ser473) (Beyotime, Shanghai, China, Cat # AF1546; 1:1000 dilution); rabbit polyclonal to c-Myc (Beyotime, Shanghai, China, Cat # AF6513; 1:1000 dilution); mouse monoclonal to E-Cadherin (Beyotime, Shanghai, China, Cat # AF0138; 1:1000 dilution); mouse monoclonal to Vimentin (Beyotime, Shanghai, China, Cat # AF0318; 1:1000 dilution)rabbit polyclonal to MMP2 (Proteintech, Wuhan, China, Cat # 10373-2-AP; 1:1000 dilution); rabbit polyclonal to MMP9 (Beyotime, Shanghai, China, Cat # AF5234; 1:1000 dilution); rabbit monoclonal to Bax (Beyotime, Shanghai, China, Cat # AF1270; 1:1000 dilution); rabbit monoclonal to Bcl-2 (Proteintech, Wuhan, China, Cat # 80313-1-RR; 1:5000 dilution). Secondary antibodies used were: HRP-labeled Goat Anti-Rabbit IgG(H + L) (Beyotime, Shanghai, China, Cat # A0208; 1:1000 dilution); HRP-labeled Goat Anti-Mouse IgG(H + L) (Beyotime, Shanghai, China, Cat # A0216; 1:1000 dilution).

### 2.4 EdU cell proliferation detection

The effect of 3-OH-B[a]A on the proliferation of breast cancer cells was evaluated using an EdU Apollo567 Cell Fluorescence *in vitro* kit. A stock solution of 3-OH-B[a]A was diluted to working concentrations of 0.4 and 2 μM in an RPMI-1640 medium supplemented with 5% activated charcoal-treated fetal bovine serum and 1% penicillin-streptomycin (double antibody). An E_2_ stock solution was diluted to a 0.5 nM working concentration using the same medium, with the final DMSO concentration maintained at ≤ 0.1% in all groups. T47D cells were pre-cultured in an RPMI-1640 medium containing 5% charcoal-stripped FBS and 1% double antibody for 24 h, then seeded into 12-well plates at a density of 1.0 × 10^5^ cells/well and incubated in a CO_2_ incubator. After 24 h of cell adhesion, the medium was replaced with fresh medium containing the aforementioned working solutions; at 68 h post-exposure, the EdU reagent was added, and the cells were further incubated for 4 h, followed by sequential procedures of cell fixation, Apollo staining, and DNA staining.

### 2.5 Colony formation assay

T47D cells were seeded into 6-well plates at a density of 400 cells/well and incubated in a CO_2_ incubator. After 24 h of culture to allow cell adhesion, the original culture medium was discarded, and the cells were treated with culture media containing the respective working solutions. The drug-containing media were refreshed every 2–3 days, and the cells were cultured for 15 consecutive days. The cells were then washed twice with PBS, fixed with 4% paraformaldehyde, and stained with crystal violet. Colonies containing more than 50 cells were counted. The experiment was performed in triplicate.

### 2.6 Scratch assay

T47D cells were seeded into 24-well plates at a density of 1.9 × 10^5^ cells/well and cultured in the CO_2_ incubator. After the cells adhered to the plate, the medium was replaced with an 1% RPMI 1640 medium for 24-h starvation. A 10 μL pipette tip was used to scratch the cell monolayer vertically from top to bottom in each well; afterward, the medium was discarded, and the cells were washed twice with PBS. Subsequently, the cells in different groups were treated with culturing media containing different working solutions, and the same position of the scratch was imaged under a microscope at 0, 12, 24, 36, and 48 h post-scratch exposure.

### 2.7 Transwell invasion assay

Matrigel was mixed with a basic medium at a ratio of 1:8, and the mixture was added to the bottom of the Transwell insert, which was then placed in the CO_2_ incubator to allow the gel to solidify. The Transwell insert was taken out, excess liquid was discarded, and an appropriate cell suspension was prepared using a basic medium containing 5 nM E_2_ and different concentrations of 3-OH-B[a]A; T47D cells were seeded into the Transwell insert at a density of 5 × 10^4^ cells/insert, while a 10% RPMI 1640 medium was added to the lower chamber, followed by incubation in the CO_2_ incubator for 72 h. After incubation, the 24-well plate was taken out, and the matrigel remaining in the upper layer of the Transwell insert was removed with a cotton swab. The insert was washed twice with PBS, fixed with 4% paraformaldehyde solution, stained with crystal violet, rinsed with ultrapure water, and then inverted and air-dried.

### 2.8 One-step TUNEL assay for apoptosis detection

The apoptotic effect of 3-OH-B[a]A on breast cancer cells was evaluated using a one-step TUNEL assay kit. Working solutions of 3-OH-B[a]A (0.4 and 2 μM) and E_2_ (0.5 nM) were prepared by diluting the respective stock solutions with the RPMI-1640 medium supplemented with 5% activated charcoal-treated fetal bovine serum and 1% penicillin/streptomycin, ensuring a final DMSO concentration of ≤ 0.1%. T47D cells were seeded in 96-well plates at a density of 1.5 × 10^4^ cells/well and cultured in the same medium for 24 h in the CO_2_ incubator to allow attachment. After 24 h, the medium was replaced with treatment media containing the respective working solutions. Following 72 h of exposure, the cells were fixed and subjected to TUNEL staining and DAPI counterstaining.

### 2.9 Western blot

For the detection of AKT, p-AKT, c-Myc, Bax, and Bcl-2 proteins, T47D cells were cultured for 24 h in the RPMI-1640 medium supplemented with 5% activated charcoal-treated fetal bovine serum and 1% double-antibody. T47D cells were seeded at a density of 6.0 × 10^5^ cells/well in 6-well plates and incubated in the CO_2_ incubator. After 24 h of cell attachment, the 3-OH-B[a]A stock solution was diluted to 0.4 and 2 μM working solutions in the RPMI-1640 medium supplemented with 5% activated charcoal-treated fetal bovine serum and 1% dual antibodies; the E_2_ stock solution was diluted to 0.5 and 5 nM working solutions. The final DMSO concentration was ≤ 0.1%. Media containing different working solutions were added. For the detection of E-Cadherin, Vimentin, MMP2, and MMP9 proteins, T47D cells were seeded at 6.0 × 10^5^ cells/well in 6-well plates and cultured in the CO_2_ incubator. After 24 h of cell attachment, the cells were starved for 24 h in an RPMI-1640 medium supplemented with 1% FBS and 1% double antibody. The 3-OH-B[a]A stock solution was diluted into 0.4 and 2 μM working solutions in the RPMI-1640 medium containing 1% FBS and 1% double antibody. The E_2_ stock solution was diluted to 0.5 and 5 nM working solutions, ensuring a final DMSO concentration of ≤ 0.1%. The respective working solutions were added to the culture medium. After 72 h exposure, cells were lysed on ice using a cell lysis buffer containing protease and phosphatase inhibitors to collect total cellular proteins. Protein concentrations were measured using the BCA assay. SDS-PAGE was performed at concentrations that were selected based on the protein molecular weight, followed by electrophoresis and membrane transfer. Blocking was performed with 5% skim milk powder or 5% BSA for 1.5 h, then it was incubated with primary antibody overnight, washed four times with 1×TBST (10 min/wash), incubated with secondary antibody for 1.5 h, washed four times with 1×TBST (10 min/wash), and finally developed.

### 2.10 Statistical analysis

One-way ANOVA was used for the analysis using GraphPad Prism 9.5 software. Data is expressed as mean ± SD. All the experiments were carried out in triplicate to ensure reproducibility.

## 3 Results

### 3.1 Proliferative effects of 3-hydroxybenz[a]anthracene in T47D cells

**FIGURE 1:**
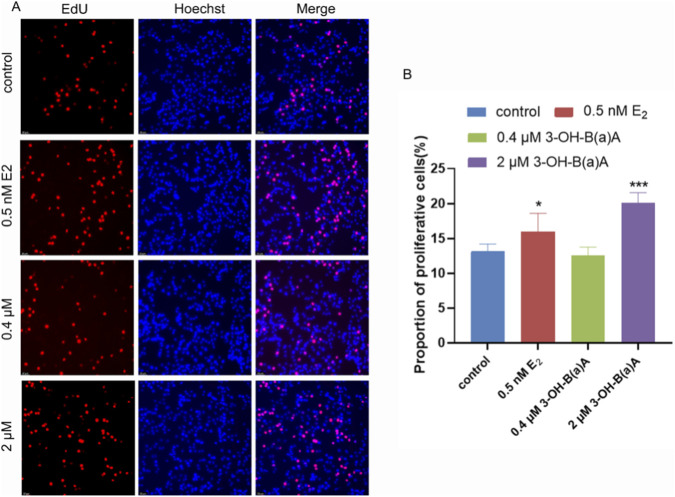
Effects of 3-OH-B[a]A and E_2_ on T47D cell proliferation after 72 h of exposure (*n* = 3). **(A)** Representative fluorescence images showing T47D cell proliferation following treatment with 3-OH-B[a]A and E_2_ for 72 h. **(B)** Quantitative comparison of the proportion of proliferative cells derived from **(A)**. Data are presented as mean ± SD. *p < 0.05 and ***p < 0.001 versus the vehicle control.

Following 72 h exposure of T47D cells to varying concentrations of 3-OH-B[a]A and 17β-estradiol (E_2_), proliferation was assessed (Figure 1). As shown in Figure 1A, treatment with 0.5 nM E_2_ or 2 μM 3-OH-B[a]A markedly increased the number of EdU-positive cells compared to the control. Quantitative analysis (Figure 1B) confirmed that the proportion of proliferative cells was significantly elevated in the 0.5 nM E_2_ and 2 μM 3-OH-B[a]A treatment groups (*p* < 0.05 or *p* < 0.001), reaching 15.95% and 20.13%, respectively. In contrast, 0.4 μM 3-OH-B[a]A did not significantly alter the proportion of proliferative cells (12.57%), a level comparable to the control. Direct comparison showed that 2 μM 3-OH-B[a]A induced an even higher proportion of proliferative cells (20.13%) than 0.5 nM E_2_, indicating potent estrogen-like proliferative activity. Collectively, these findings indicate that 3-OH-B[a]A at 2 μM significantly enhances the proliferative capacity of T47D cells.

### 3.2 Colony-promoting effects of 3-hydroxybenz[a]anthracene in T47D cells

**FIGURE 2:**
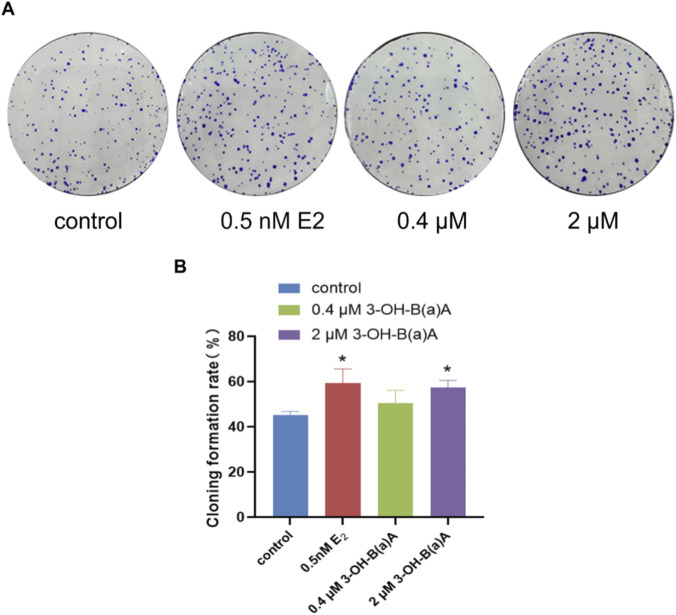
Effects of 3-OH-B[a]A and E_2_ on colony formation of T47D cells after 15 days of exposure (*n* = 3). **(A)** Representative images of T47D cell colonies following treatment with 3-OH-B[a]A and E_2_ for 15 days. **(B)** Quantitative analysis of colony formation rate derived from **(A)**. Data are presented as mean ± SD. *p < 0.05 versus the vehicle control.

The effects of 15-day exposure to varying concentrations of 3-OH-B[a]A and E_2_ on the clonogenic potential of T47D cells are presented in Figure 2. As illustrated in Figure 2A, treatment with 0.5 nM E_2_ or 2 μM 3-OH-B[a]A resulted in a marked increase in the number of colonies formed compared with the control group. Quantitative evaluation shown in Figure 2B further confirmed that colony formation rates were 59.38% in the 0.5 nM E_2_ group, 50.50% in the 0.4 μM 3-OH-B[a]A group, and 57.42% in the 2 μM 3-OH-B[a]A group. Both the 0.5 nM E_2_ and 2 μM 3-OH-B[a]A groups showed significantly elevated colony formation compared to the control (*p* < 0.05). Notably, 2 μM 3-OH-B[a]A exhibited colony-forming activity comparable to, though slightly lower than, that of 0.5 nM E_2_, whereas 0.4 μM 3-OH-B[a]A did not induce any significant change in clonogenic capacity. Collectively, these findings indicate that exposure to 3-OH-B[a]A at 2 μM significantly enhances the clonogenic potential of T47D cells.

### 3.3 Migratory effects of 3-hydroxybenz[a]anthracene in T47D cells

**FIGURE 3:**
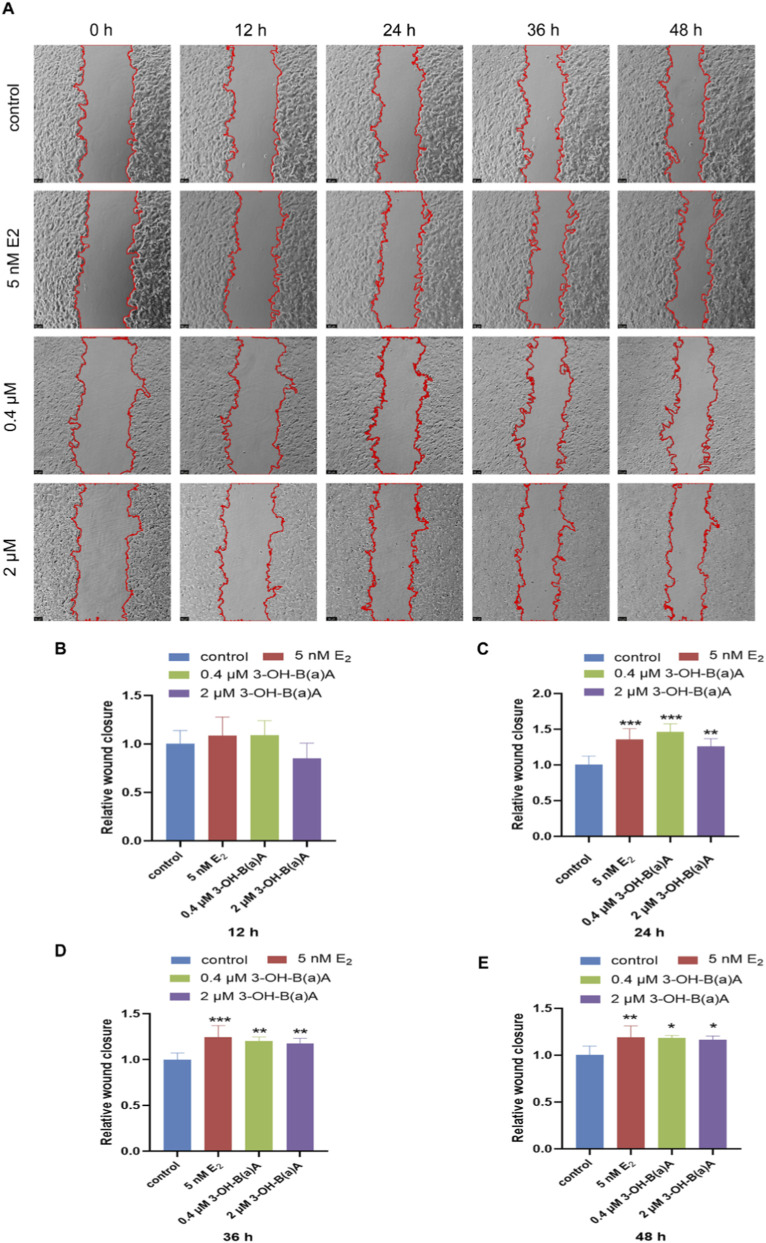
Effects of 3-OH-B[a]A and E_2_ on the migration of T47D cells over 0–48 h (*n* = 3). **(A)** Representative images showing T47D cell migration following treatment with 3-OH-B[a]A and E_2_ for 0, 12, 36, and 48 h **(B–E)** Quantitative comparison of the relative migration area derived from **(A)**. Data are presented as mean ± SD. *p < 0.05, **p < 0.01, and ***p < 0.001 versus the vehicle control.

The migratory behavior of T47D cells following exposure to varying concentrations of 3-OH-B[a]A and E_2_ over 0, 12, 24, 36, and 48 h is presented in Figure 3. As shown in Figures 3C–E, treatment with 5 nM E_2_, 0.4 μM, or 2 μM 3-OH-B[a]A resulted in significantly increased relative migration areas compared with the control group at 24 h, 36 h, and 48 h (*p* < 0.05, *p* < 0.01, or *p* < 0.001), reaching 1.36, 1.46 and 1.26 (24 h), 1.25, 1.20 and 1.18 (36 h), and 1.19, 1.19 and 1.17 (48 h), respectively. The relative migration area in the 0.4 μM and 2 μM 3-OH-B[a]A-treated groups peaked at 24 h. Consistent with these findings, Figure 3B shows that no significant differences in relative migration area were observed among any treatment groups following 12 h of exposure. Collectively, these findings demonstrate that exposure to 0.4 μM and 2 μM 3-OH-B[a]A markedly enhances the migration capacity of T47D cells, with the maximal effect observed at 24 h.

### 3.4 Invasive effects of 3-hydroxybenz[a]anthracene in T47D cells

**FIGURE 4:**
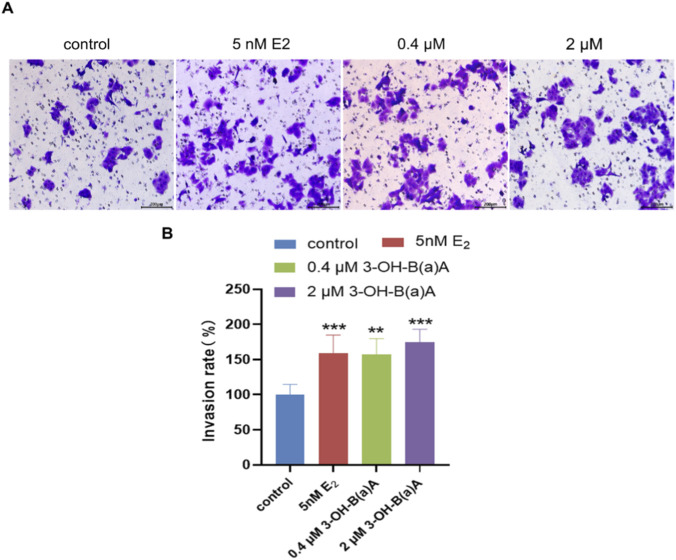
Effects of 3-OH-B[a]A and E_2_ on the invasion of T47D cells after 72 h of exposure (*n* = 3) **(A)**. Representative images showing T47D cell invasion following treatment with 3-OH-B[a]A and E_2_ for 72 h **(B)**. Quantitative analysis of the invasion rate derived from **(A)**. Data are presented as mean ± SD. **p < 0.01 and ***p < 0.001 versus the vehicle control.

Figure 4 illustrates the invasive response of T47D cells following 72 h of exposure to varying concentrations of 3-OH-B[a]A and E_2_. As shown in Figure 4A, a marked increase in the number of cells penetrating the polyester membrane is observed after 72 h of exposure to 5 nM E_2_, 0.4 μM 3-OH-B[a]A, and 2 μM 3-OH-B[a]A compared with the untreated control. Quantitative analysis (Figure 4B) confirmed that all three treatments significantly enhance the invasion rate of T47D cells (*p* < 0.01 or *p* < 0.001), with invasion rates reaching 158.55% for 5 nM E_2_, 156.97% for 0.4 μM 3-OH-B[a]A, and 174.93% for 2 μM 3-OH-B[a]A relative to the control (set as 100%). Notably, the highest invasion rate (174.93%) is achieved with 2 μM 3-OH-B[a]A treatment, while the invasion rates induced by 5 nM E_2_ and 0.4 μM 3-OH-B[a]A are comparable. Collectively, these findings demonstrate that both 0.4 μM and 2 μM 3-OH-B[a]A significantly promote the invasive capacity of T47D cells.

### 3.5 Apoptotic effects of 3-hydroxybenz[a]anthracene in T47D cells

**FIGURE 5:**
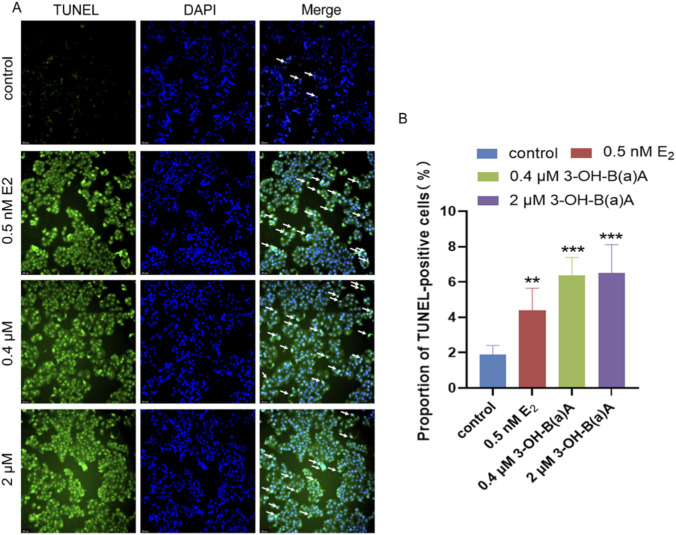
Effects of 3-OH-B[a]A and E_2_ on apoptosis of T47D cells after 72 h of exposure (*n* = 3) **(A)**. Representative images showing apoptosis of T47D cells following treatment with 3-OH-B[a]A and E_2_ for 72 h, as assessed by the TUNEL assay. Arrows indicate TUNEL-positive apoptotic cells **(B)**. Quantitative analysis of the proportion of TUNEL-positive cells derived from **(A)**. Data are presented as mean ± SD. **p < 0.01 and ***p < 0.001 versus the vehicle control.

Figure 5 depicts the apoptotic response of T47D cells following 72 h of exposure to varying concentrations of 3-OH-B[a]A and E_2_. As shown in Figure 5A, a marked increase in TUNEL-positive cells was observed in cells treated with 0.5 nM E_2_, 0.4 μM, and 2 μM 3-OH-B[a]A relative to the untreated control. Quantitative analysis (Figure 5B) revealed that the proportion of apoptotic cells was 1.88% in the control group, 4.39% in the 0.5 nM E_2_ group, 6.36% in the 0.4 μM 3-OH-B[a]A group, and 6.52% in the 2 μM 3-OH-B[a]A group. All three treatments significantly promoted apoptotic activity (*p* < 0.01 or *p* < 0.001). The highest proportion of apoptotic cells (6.52%) was achieved with 2 μM 3-OH-B[a]A, followed by 0.4 μM 3-OH-B[a]A (6.36%). Collectively, these findings demonstrate that both 0.4 μM and 2 μM 3-OH-B[a]A significantly enhance the apoptotic capacity of T47D cells.

### 3.6 Modulation of protein expression by 3-OH-B[a]A in T47D cells

**FIGURE 6:**
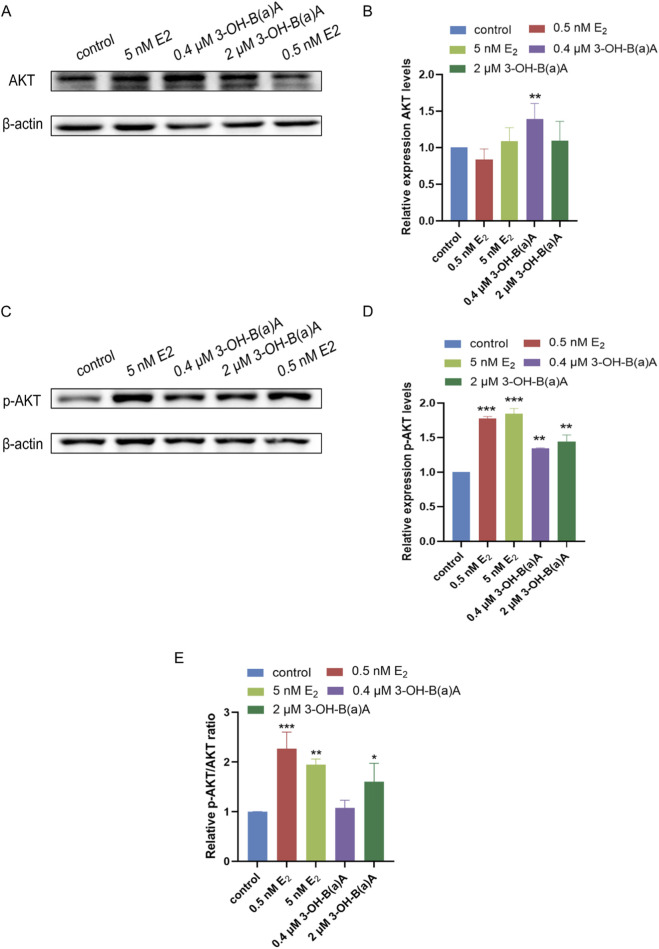
Effect of 3-OH-B[a]A and E_2_ on the expression of AKT, p-AKT, and p-AKT/AKT ratio in T47D cells (*n* = 3). **(A)** Representative immunoblot showing AKT expression in T47D cells following treatment with 3-OH-B[a]A and E_2_. **(B)** Quantitative analysis of relative AKT protein expression corresponding to **(A,C)** Representative immunoblot showing p-AKT expression. **(D)** Quantitative analysis of relative p-AKT expression corresponding to **(C,E)** The p-AKT/AKT ratio is expressed as fold change relative to the control group (set as 1). Data are presented as mean ± SD. *p < 0.05, **p < 0.01, and ***p < 0.001 versus the vehicle control.

Exposure of T47D cells to varying concentrations of 3-OH-B[a]A and E_2_ for 72 h resulted in significant changes in the expression levels of selected proteins. Figure 6 presents Western blot analyses evaluating the effects of 3-OH-B[a]A and E_2_ on the expression of AKT, phosphorylated AKT (p-AKT), and p-AKT/AKT ratio in T47D cells. As shown in Figures 6A,B, treatment with E_2_ at concentrations of 0.5 nM and 5 nM did not produce any significant alteration in total AKT protein levels relative to the control (relative expression: 0.83 and 1.09, respectively). In contrast, exposure to 0.4 μM 3-OH-B[a]A resulted in a significant upregulation of AKT protein expression (1.39-fold compared with the control), whereas treatment with 2 μM 3-OH-B[a]A produced no statistically significant change (relative expression: 1.10), reflecting the response observed in the E_2_-treated groups. Collectively, these findings demonstrate that 3-OH-B[a]A at 0.4 µM selectively enhances AKT protein expression in human breast cancer T47D cells.

As shown in Figures 6C,D, treatment with E_2_ at concentrations of 0.5 nM and 5 nM significantly enhanced p-AKT protein expression relative to the control, with relative expression levels of 1.77 and 1.85, respectively. Similarly, exposure to 0.4 μM and 2 μM 3-OH-B[a]A notably increased the level of p-AKT protein (1.34 and 1.44, respectively), consistent with the effects observed in the E_2_-treated groups. Collectively, these data demonstrate that 3-OH-B[a]A at 0.4 μM and 2 μM enhances AKT phosphorylation in human breast cancer T47D cells.

To further assess AKT pathway activation, we calculated the p-AKT/AKT ratio based on densitometric analysis (Figure 6E). Compared to the control group, 2 μM 3-OH-B[a]A significantly increased the p-AKT/AKT ratio (*p* < 0.05), while 0.4 μM 3-OH-B[a]A showed a trend toward elevation but did not reach statistical significance. As positive controls, 0.5 nM and 5 nM E_2_ both markedly increased the p-AKT/AKT ratio (*p* < 0.001 and *p* < 0.01, respectively). These results indicated that 2 μM 3-OH-B[a]A enhanced AKT phosphorylation, consistent with its estrogen-like activity, whereas the effect of 0.4 μM 3-OH-B[a]A on this ratio was not statistically significant despite its impact on total AKT expression.

**FIGURE 7:**
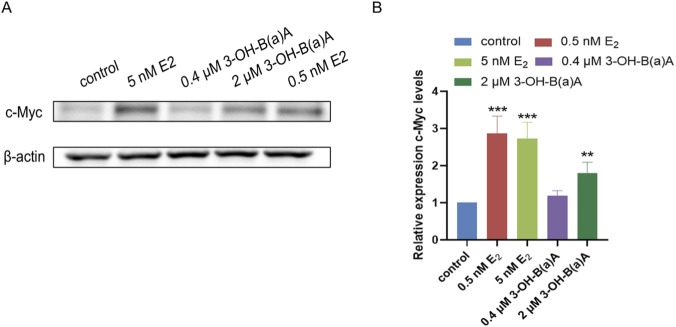
Effect of 3-OH-B[a]A and E_2_ on the expression of c-Myc in T47D cells (*n* = 3). **(A)** Representative immunoblot showing c-Myc expression in T47D cells following treatment with 3-OH-B[a]A and E_2_. **(B)** Quantitative analysis of relative c-Myc protein expression corresponding to **(A)**. Data are presented as mean ± SD. **p < 0.01 and ***p < 0.001 versus the vehicle control.

Western blot analysis assessing the effects of 3-OH-B[a]A and E_2_ on T47D cells revealed the expression profiles of c-Myc (Figure 7). As illustrated in Figures 7A,B, exposure to E_2_ at 0.5 nM and 5 nM significantly increased c-Myc expression relative to the control, with relative expression levels of 2.87 and 2.74, respectively. Consistent with this trend, treatment with 2 μM 3-OH-B[a]A also markedly upregulated c-Myc levels (1.81), whereas exposure to 0.4 μM of the compound produced no statistically significant change (1.19). Collectively, these data demonstrate that 3-OH-B[a]A at 2 μM enhances c-Myc protein expression in human breast cancer T47D cells.

**FIGURE 8:**
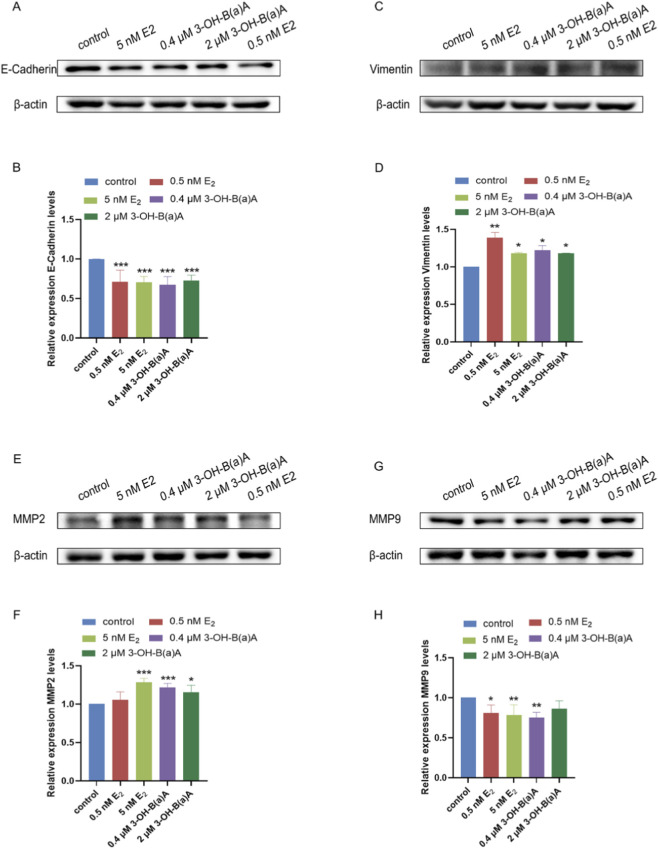
Effects of 3-OH-B[a]A and E_2_ on the expression of E-Cadherin, Vimentin, MMP2, and MMP9 in T47D cells (*n* = 3). **(A)** Representative immunoblot showing E-Cadherin expression following treatment with 3-OH-B[a]A and E_2_. **(B)** Quantitative analysis of relative E-Cadherin protein expression corresponding to **(A,C)** Representative immunoblot showing Vimentin expression. **(D)** Quantitative analysis of relative Vimentin protein expression corresponding to **(C,E)** Representative immunoblot showing MMP2 expression. **(F)** Quantitative analysis of relative MMP2 protein expression corresponding to **(E,G)** Representative immunoblot showing MMP9 expression. **(H)** Quantitative analysis of relative MMP9 protein expression corresponding to **(G)**. Data are presented as mean ± SD. *p < 0.05, **p < 0.01, and ***p < 0.001 versus the vehicle control.

Western blot analysis assessing the effects of 3-OH-B[a]A and E_2_ on T47D cells revealed the expression profiles of E-Cadherin, Vimentin, MMP2, and MMP9 (Figure 8). As shown in Figures 8A,B, treatment with E_2_ at 0.5 nM and 5 nM significantly downregulated E-Cadherin expression relative to the control, with relative expression levels of 0.71 and 0.70, respectively. Similarly, exposure to 0.4 μM and 2 μM 3-OH-B[a]A resulted in a marked reduction of E-Cadherin protein levels (0.69 and 0.73, respectively). Collectively, these findings indicate that 3-OH-B[a]A at 0.4 μM and 2 μM downregulates E-Cadherin expression in human breast cancer T47D cells.

As illustrated in Figures 8C,D, treatment with 0.5 nM and 5 nM E_2_ resulted in a significant increase in Vimentin expression compared with the control, with relative expression levels of 1.39 and 1.18, respectively. Similarly, exposure to 0.4 μM and 2 μM 3-OH-B[a]A markedly upregulated Vimentin protein levels (1.22 and 1.18, respectively). Collectively, these findings indicate that 3-OH-B[a]A at 0.4 μM and 2 μM upregulates Vimentin expression in human breast cancer T47D cells.

As shown in 8E,F, exposure to 5 nM E_2_ significantly increased MMP2 expression relative to the control (1.28), whereas treatment with 0.5 nM E_2_ did not result in a statistically significant change (1.06). Similarly, T47D cells treated with both 0.4 μM and 2 μM 3-OH-B[a]A exhibited a marked upregulation of MMP2 levels, with relative expression values of 1.22 and 1.16, respectively. Collectively, these findings demonstrate that 3-OH-B[a]A at 0.4 μM and 2 μM upregulates MMP2 protein expression in human breast cancer T47D cells.

As shown in Figures 8G,H, treatment with E_2_ at 0.5 nM and 5 nM significantly decreased MMP9 expression compared with the control, with relative expression levels of 0.81 and 0.78, respectively. Similarly, exposure of T47D cells to 0.4 μM 3-OH-B[a]A resulted in a significant downregulation of MMP9 levels (0.75), whereas treatment with 2 μM of the compound did not induce a statistically significant change (0.86). Collectively, these findings indicate that exposure to 3-OH-B[a]A at 0.4 µM significantly downregulates MMP9 protein expression in human breast cancer T47D cells.

**FIGURE 9:**
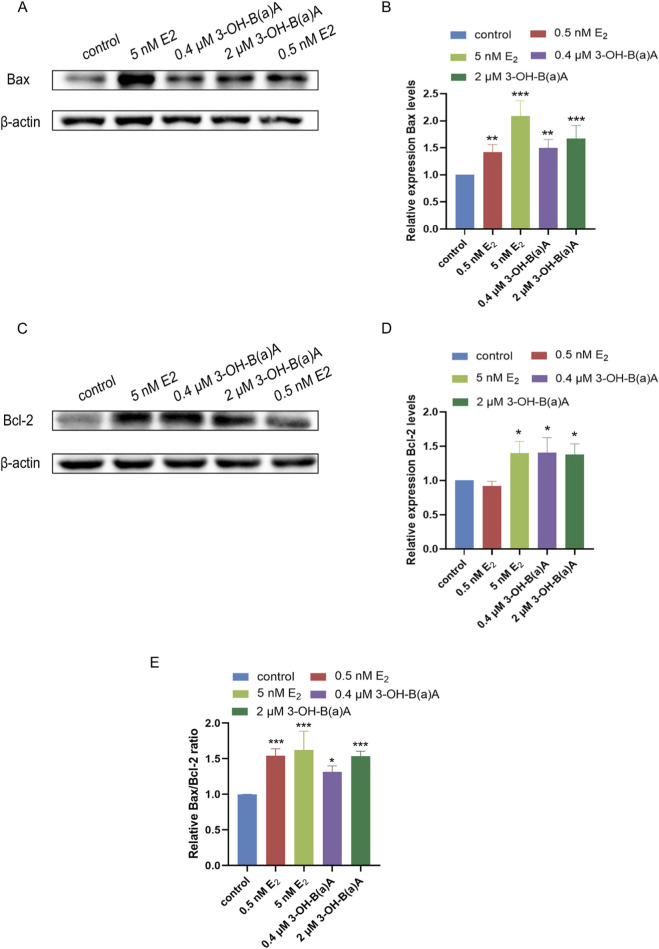
Effect of 3-OH-B[a]A and E_2_ on the expression of apoptosis-related proteins Bax, Bcl-2, and Bax/Bcl-2 ratio in T47D cells (*n* = 3). **(A)** Representative immunoblot showing Bax expression following treatment with 3-OH-B[a]A and E_2_. **(B)** Quantitative analysis of relative Bax protein expression corresponding to **(A,C)** Representative immunoblot showing Bcl-2 expression. **(D)** Quantitative analysis of relative Bcl-2 protein expression corresponding to **(C,E)** Quantitative analysis of the Bax/Bcl-2 ratio in T47D cells after treatment with 3-OH-B[a]A and E2. The Bax/Bcl-2 ratio is expressed as fold change relative to the control group (set as 1). Data are presented as mean ± SD. *p < 0.05, **p < 0.01, and ***p < 0.001 versus the vehicle control.

Figure 9 presents Western blot analyses examining the effects of 3-OH-B[a]A and E_2_ on the expression of the apoptosis-related proteins Bax, Bcl-2, and Bax/Bcl-2 ratio in T47D cells. As shown in Figures 9A,B, treatment with 0.5 nM and 5 nM E_2_ resulted in a significant upregulation of Bax protein expression relative to the control, with relative expression levels of 1.42 and 2.09, respectively. Similarly, exposure to 0.4 μM and 2 μM 3-OH-B[a]A markedly increased Bax levels (1.50 and 1.67, respectively). Collectively, these findings demonstrate that 3-OH-B[a]A at 0.4 μM and 2 μM upregulates Bax protein expression in human breast cancer T47D cells.

As shown in Figures 9C,D, treatment with 5 nM E_2_ resulted in a significant upregulation of Bcl-2 protein expression relative to the control, with relative expression levels of 1.40, whereas treatment with 0.5 nM E_2_ did not result in a statistically significant change. Similarly, exposure to 0.4 μM and 2 μM 3-OH-B[a]A markedly increased Bcl-2 levels (1.41 and 1.38, respectively). Collectively, these findings demonstrate that 3-OH-B[a]A at 0.4 μM and 2 μM upregulates Bcl-2 protein expression in human breast cancer T47D cells.

Figure 9E showed that despite increased Bcl-2 expression, the Bax/Bcl-2 ratio was significantly elevated in both 3-OH-B[a]A-treated groups relative to the control, reaching levels comparable to the pro-apoptotic effect of 0.5 nM E_2_. This confirmed that 3-OH-B[a]A shifted the BCL-2 family balance towards apoptosis via BCL-2 family regulation.

## 4 Discussion

In this study, a series of experiments was conducted for the first time to evaluate the estrogen-like effects of the PAH derivative 3-OH-B[a]A on the ER-positive T47D breast cancer cell line and its malignant phenotypes. The obtained results indicate that 3-OH-B[a]A significantly enhances estrogen activity in T47D cells, thereby promoting cell proliferation, migration, and invasion while simultaneously inducing apoptosis. It was previously reported that estrogenic compounds promoted the malignant progression of breast cancer cells. For example, the environmental endocrine-disrupting chemicals (EDCs) like bisphenol A and dodecafluoro-1,6-diiodohexane significantly enhanced the proliferation of estrogen receptor-positive MCF-7 and T47D breast cancer cells (Lloyd, et al., 2019; Song, et al., 2018). Treatment with triclosan and bisphenol A induced anti-apoptosis in VM7Luc4E2 breast cancer cells (Lee, et al., 2018), while bisphenol A and nonylphenol (NP) have the potential to promote epithelial-mesenchymal transition (EMT) and migration of estrogen-responsive cancers (Kim, et al., 2015). Long-term exposure to 10^−7^ M of triclosan increased the migration and invasion of estrogen-responsive MCF-7 breast cancer cells (after 17 weeks) (Farasani and Darbre, 2021). All these studies demonstrate an increased risk of breast cancer development following exposure to estrogenic endocrine disruptors.

We investigated whether 3-OH-B[a]A influences T47D breast cancer cell proliferation via the PI3K/AKT signaling pathway. Based on the performed EdU fluorescence experiments and cell colony formation assays, 3-OH-B[a]A was observed to promote T47D cell proliferation. The PI3K/AKT pathway is abnormally activated in multiple cancers, promoting tumor growth and metastasis. For instance, in thyroid cancer, the STAT3-induced lncRNA ABHD11-AS1 promotes progression by regulating this pathway (Wen et al., 2019), while in lung adenocarcinoma, GPM6A upregulation suppresses the PI3K/AKT pathway to reduce cell proliferation (Zhang et al., 2022). In colorectal cancer, SPAG5 promotes malignant phenotypes by activating PI3K/AKT (Zhang X. et al., 2023), while DMC-BH exerts anticancer effects by inhibiting this pathway (Liu et al., 2023). The effects of 3-OH-B[a]A on the expression of key proteins in the PI3K/AKT pathway were studied. The results in Figure 6 showed that 3-OH-B[a]A significantly upregulates AKT and p-AKT expression, suggesting that it may promote cell proliferation by activating the PI3K/AKT pathway. It is well documented that estrogen primarily regulates AKT activity through rapid phosphorylation rather than altering total AKT protein expression (Guo et al., 2006; Lee et al., 2005). The p-AKT/AKT ratio is therefore the standard measure of pathway activation (Guo et al., 2006). Consistent with this canonical mechanism, our results showed that 0.5 nM and 5 nM E_2_ increased p-AKT levels without significantly affecting total AKT expression (Figure 6), further supporting the validity of our experimental system. As shown in Figure 6E, 2 μM 3-OH-B[a]A significantly elevated the p-AKT/AKT ratio compared with the control, suggesting enhanced AKT phosphorylation. These findings further validated the pro-proliferative effect of 3-OH-B[a]A in T47D cells. Additionally, 3-OH-B[a]A significantly increased the expression of the transcription regulator c-Myc, which plays a pivotal role in tumorigenesis and progression, regulating processes including cell proliferation, cell cycle progression, and apoptosis (Jiang et al., 2022; Vita and Henriksson, 2006). This finding further supported the pro-proliferative effect of 3-OH-B[a]A on the T47D breast cancer cell line.

Metastasis is the leading cause of cancer-related deaths worldwide, representing a complex process involving multiple steps such as cancer cell dissemination, invasion, migration, hematogenous spread, hemorrhage, and implantation (Saxena et al., 2020). EMT serves as a key driving mechanism, playing a critical role in cancer progression by conferring migratory and invasive capabilities to cancer cells (Mittal, 2018; Saxena et al., 2020). Based on the conducted wound healing assays that indicated that 3-OH-B[a]A may promote T47D cell migration, we examined its effects on the expression of key EMT markers. 3-hydroxy-B(a) significantly downregulated the expression of the epithelial marker protein E-Cadherin and simultaneously upregulated the expression of the mesenchymal marker protein Vimentin. The reduced E-Cadherin expression and the increased Vimentin expression are hallmark features of EMT, and similar phenomena were reported in other tumor models, such as prostate cancer (Jiang, 2025). These changes in protein levels are well-correlated with the enhanced cell migration capacity, suggesting that 3-OH-B[a]A may promote breast cancer cell migration by inducing the EMT process.

Local invasion of tumors into adjacent tissues is regulated by the coordinated action of multiple signaling pathways, including alterations in tumor cell cytoskeletal dynamics, extracellular matrix, and cell adhesion properties (Friedl and Alexander, 2011). During this process, matrix metalloproteinases (MMPs) promote tumor invasion and metastasis by degrading the components of the extracellular matrix (Kawai et al., 2021). Based on the performed Transwell invasion assays that suggested that 3-OH-B[a]A may promote T47D cell invasion, the effects of 3-OH-B[a]A on MMP2 and MMP9 protein expression were studied, indicating that 3-OH-B[a]A significantly upregulated MMP2 and downregulated MMP9 expression. Although MMP9 was downregulated, invasion was significantly enhanced. This discrepancy may be attributed to the upregulation of E-Cadherin, which counteracts the reduction in invasiveness caused by MMP9 downregulation. It may reflect compensatory upregulation of other MMPs (Akhlaghipour and Moghbeli, 2024), altered TIMP expression shifting the proteolytic balance (Bourboulia and Stetler-Stevenson, 2010), or adoption of MMP-independent migration modes (Vérollet et al., 2011). The expressions of MMP2 and MMP9 are often regulated by distinct signaling pathways. MMP2 expression is closely associated with the activation of the TGF-β signaling pathway, while MMP2 deficiency can inhibit the activity of this pathway (Lian et al., 2021). In contrast, MMP9 expression is frequently positively regulated by the NF-κB signaling pathway; inhibiting NF-κB reduces MMP-9 expression and attenuates cell invasion (Kim et al., 2012). Therefore, 3-OH-B[a]A may promote MMP2 expression by activating the TGF-β pathway while simultaneously reducing MMP9 expression through inhibition of the NF-κB pathway, thereby jointly influencing cellular invasion capacity. However, this hypothesis requires further experimental validation.

Apoptosis is a strictly regulated programmed cell death process that plays a crucial role in maintaining tissue homeostasis and eliminating abnormal cells (Galluzzi et al., 2018). This process is primarily regulated by the B-cell lymphoma 2 (BCL-2) protein family, which includes both pro-apoptotic proteins like Bax and anti-apoptotic proteins like Bcl-2. The cellular equilibrium between pro-apoptotic and anti-apoptotic proteins determines whether a cell survives or undergoes death (Sato et al., 2022). Based on the conducted TUNEL apoptosis assays, which suggested that 3-OH-B[a]A may induce apoptosis in T47D cells, we further examined the effects of 3-OH-B[a]A on Bax and Bcl-2 protein expression. The concurrent upregulation of both Bax and Bcl-2 by 3-OH-B[a]A may seem contradictory. This dual regulatory pattern may be attributed to the activation of multiple signaling pathways by the compound: the PI3K/AKT/mTOR signaling pathway mediates apoptosis inhibition (Zhou et al., 2024), whereas stress-responsive pathways elevate Bax levels (Steckley et al., 2007). Despite this, the net effect should be determined by the Bax/Bcl-2 ratio, which dictates mitochondrial outer-membrane permeabilization. The Bax/Bcl-2 ratio, rather than the absolute levels, is a critical determinant of apoptosis. Notably, quantitative analysis of the ratio (Figure 9E) revealed marked increases in both 3-OH-B[a]A-treated groups and the two positive control groups relative to the control group, which further verified the transition to a pro-apoptotic phenotype (Figure 5). Similar findings were reported in the literature, for example, E_2_ exhibited dual regulatory effects on MCF-7 cell apoptosis (Zhou et al., 2024). Although the levels of Bax and Bcl-2 increased in this study, cells ultimately underwent apoptosis, suggesting that pro-apoptotic signals may dominate or that other apoptotic mechanisms were activated.

Although 3-OH-B[a]A simultaneously promoted proliferation, migration, and invasion while inducing apoptosis in T47D cells, these findings are not necessarily contradictory. The significantly elevated Bax/Bcl-2 ratio (Figure 9E) indicates a net shift toward pro-apoptotic signaling despite increased Bcl-2 expression. This is consistent with the known ability of estrogen-like compounds to concurrently activate proliferative pathways and stress-induced apoptotic checkpoints. Sustained hyperactivation of proliferative signals may trigger intrinsic apoptotic responses as a cellular protective mechanism (Fearnhead, 2004; Jordan, 2015; Yang et al., 2007), a phenomenon observed in certain endocrine-related contexts. The observed apoptosis therefore reflects an intrinsic regulatory response that does not negate the overall pro-proliferative and pro-migratory effects of 3-OH-B[a]A.

In summary, we confirmed a distinct estrogen-like activity of 3-OH-B[a]A exhibits *in vitro*, regulating malignant phenotypes such as proliferation, migration, invasion, and apoptosis in breast cancer cells by modulating the PI3K/AKT signaling pathway, EMT processes, and apoptosis-related protein expression. These findings provide experimental evidence for deepening the understanding of the endocrine-disrupting effects of PAH derivatives and their role in breast cancer initiation and progression. They also offer reference values for the risk assessment and prevention of related environmental pollutants. It should be noted that the concentrations used in this study (0.4 and 2 μM) exceed reported human urinary levels of hydroxylated PAH metabolites. Biomonitoring data indicate that metabolites of high molecular weight PAHs such as benz[a]anthracene are detected in less than 5% of the general population, with concentrations typically in the low nanomolar range (Li et al., 2008). The doses were selected based on preliminary experiments to establish clear dose-responses. While 0.4 μM may approach the upper limits of environmental exposure, 2 μM represents a supra-physiological condition. These findings provide mechanistic insights, but future studies using environmentally relevant concentrations are warranted. As the present study is solely based on *in vitro* experiments, further validation using *in vivo* models, such as breast cancer xenograft models, is necessary to establish a definite conclusion on the estrogenic activity of 3-OH-B[a]A on breast cancer. A limitation of this study is the use of a single ER-positive breast cancer cell line (T47D). While this model is appropriate for investigating estrogen-like activities, the inclusion of ER-negative breast cancer cell lines in future studies would help determine whether the observed effects—including proliferation, migration, invasion, and apoptosis modulation—are specifically ER-dependent or represent general cellular responses independent of ER status. Such comparative approaches would provide more definitive evidence for the role of ER signaling in mediating the actions of 3-OH-B[a]A.

It should also be noted that apoptosis was assessed primarily by TUNEL staining, which detects DNA fragmentation—a characteristic feature of apoptosis—but may also label cells undergoing other forms of cell death. Although the observed changes in Bax/Bcl-2 ratio support a pro-apoptotic shift, future studies employing additional apoptosis validation methods such as Annexin V/PI flow cytometry, caspase-3/7 activity assays, or PARP cleavage analysis would provide more definitive evidence.

## Data Availability

The raw data supporting the conclusions of this article will be made available by the authors, without undue reservation.

## References

[B1] Akhlaghipour I. Moghbeli M. (2024). Matrix metalloproteinases as the critical regulators of cisplatin response and tumor cell invasion. Eur. J. Pharmacol. 982, 176966. 10.1016/j.ejphar.2024.176966 39216742

[B2] Amadou A. Praud D. Coudon T. Deygas F. Grassot L. Faure E. (2021). Risk of breast cancer associated with long-term exposure to benzo a pyrene (BaP) air pollution: evidence from the French E3N cohort study. Environ. Int. 149, 106399. 10.1016/j.envint.2021.106399 33503556

[B3] Bourboulia D. Stetler-Stevenson W. G. (2010). Matrix metalloproteinases (MMPs) and tissue inhibitors of metalloproteinases (TIMPs): positive and negative regulators in tumor cell adhesion. Semin. Cancer Biol. 20 (3), 161–168. 10.1016/j.semcancer.2010.05.002 20470890 PMC2941566

[B4] Cheng M. H. Tan Z. Zeng X. W. Liu Z. Liu P. Ali A. (2023). Contamination and health risk assessment of polycyclic aromatic hydrocarbons in seasoning flour products in Hunan, China. Int. J. Environ. Res. Public Health 20 (2), 963. 10.3390/ijerph20020963 36673717 PMC9859540

[B5] Farasani A. Darbre P. D. (2021). Long-term exposure to triclosan increases migration and invasion of human breast epithelial cells *in vitro* . J. Appl. Toxicol. 41 (7), 1115–1126. 10.1002/jat.4097 33171535 PMC8246770

[B6] Fearnhead H. O. (2004). Getting back on track, or what to do when apoptosis is de-railed: recoupling oncogenes to the apoptotic machinery. Cancer Biol. Ther. 3 (1), 21–28. 10.4161/cbt.3.1.538 14726669

[B7] Friedl P. Alexander S. (2011). Cancer invasion and the microenvironment: plasticity and reciprocity. Cell. 147 (5), 992–1009. 10.1016/j.cell.2011.11.016 22118458

[B8] Galluzzi L. Vitale I. Aaronson S. A. Abrams J. M. Adam D. Agostinis P. (2018). Molecular mechanisms of cell death: recommendations of the nomenclature committee on cell death 2018. Cell. Death Differ. 25 (3), 486–541. 10.1038/s41418-017-0012-4 29362479 PMC5864239

[B9] Gündel J. Angerer J. (2000). High-performance liquid chromatographic method with fluorescence detection for the determination of 3-hydroxybenzo[a]pyrene and 3-hydroxybenz[a]anthracene in the urine of polycyclic aromatic hydrocarbon-exposed workers. J. Chromatogr. B Biomed. Sci. Appl. 738 (1), 47–55. 10.1016/s0378-4347(99)00499-5 10778925

[B10] Guo R. X. Wei L. H. Tu Z. Sun P. M. Wang J. L. Zhao D. (2006). 17 beta-estradiol activates PI3K/Akt signaling pathway by estrogen receptor (ER)-Dependent and ER-independent mechanisms in endometrial cancer cells. J. Steroid Biochem. Mol. Biol. 99 (1), 9–18. 10.1016/j.jsbmb.2005.11.013 16567092

[B11] Hayakawa K. Onoda Y. Tachikawa C. Yoshita M. Toriba A. Kameda T. (2008). Interaction of hydroxylatrd polycyclic aromatic hydrocarbons to estrogen receptor. Polycycl. Aromat. Compd. 28 (4-5), 382–391. 10.1080/10406630802374556

[B12] Honda M. Hayakawa K. Zhang L. L. Tang N. Nakamura H. (2022). Seasonal variability and risk assessment of atmospheric polycyclic aromatic hydrocarbons and hydroxylated polycyclic aromatic hydrocarbons in kanazawa, Japan. Appl. Sciences-Basel 12 (19), 9469. 10.3390/app12199469

[B13] Hwang M. J. Kang S. J. Kim H. S. Lee K. W. (2019). Reduction of the polycyclic aromatic hydrocarbon levels in dried red peppers (Capsicum annuum L.) using heat pump-assisted drying. Food Chem. 297, 124977. 10.1016/j.foodchem.2019.124977 31253260

[B14] Jiang H. (2025). Prostate cancer bone metastasis: molecular mechanisms of tumor and bone microenvironment. Cancer Manag. Res. 17, 219–237. 10.2147/cmar.S495169 39912095 PMC11796448

[B15] Jiang T. Yang J. H. Yang H. H. Chen W. Ji K. Xu Y. (2022). SLC35B4 stabilizes c-MYC protein by O-GlcNAcylation in HCC. Front. Pharmacol. 13, 851089. 10.3389/fphar.2022.851089 35308201 PMC8924407

[B16] Jordan V. C. (2015). The new biology of estrogen-induced apoptosis applied to treat and prevent breast cancer. Endocr. Relat. Cancer 22 (1), R1–R31. 10.1530/erc-14-0448 25339261 PMC4494663

[B17] Ju Y. R. Chen C. F. Wang M. H. Dong C. D. (2022). Assessment of polycyclic aromatic hydrocarbons in seafood collected from coastal aquaculture ponds in Taiwan and human health risk assessment. J. Hazard. Mater. 421, 126708. 10.1016/j.jhazmat.2021.126708 34352521

[B33] Kawai H. Takabatake K. Shan Q. Eain H. S. Sukegawa S. (2021). Cancer-associated stromal cells promote the contribution of MMP2-positive bone marrow-derived cells to oral squamous cell carcinoma invasion. Cancers (Basel) 14 (1), 137. 10.3390/cancers14010137 35008304 PMC8750016

[B18] Kim J. H. Kim M. S. Bak Y. Chung I. M. Yoon D. Y. (2012). The cadin-2-en-1β-ol-1β-D-glucuronopyranoside suppresses TPA-Mediated matrix metalloproteinase-9 expression through the ERK signaling pathway in MCF-7 human breast adenocarcinoma cells. J. Pharmacol. Sci. 118 (2), 198–205. 10.1254/jphs.11196FP 22293298

[B19] Kim Y. S. Choi K. C. Hwang K. A. (2015). Genistein suppressed epithelial-mesenchymal transition and migration efficacies of BG-1 ovarian cancer cells activated by estrogenic chemicals *via* estrogen receptor pathway and downregulation of TGF-β signaling pathway. Phytomedicine 22 (11), 993–999. 10.1016/j.phymed.2015.08.003 26407941

[B20] Lee Y. R. Park J. Yu H. N. Kim J. S. Youn H. J. Jung S. H. (2005). Up-regulation of PI3K/Akt signaling by 17beta-estradiol through activation of estrogen receptor-alpha, but not estrogen receptor-beta, and stimulates cell growth in breast cancer cells. Biochem. Biophys. Res. Commun. 336 (4), 1221–1226. 10.1016/j.bbrc.2005.08.256 16169518

[B21] Lee G. A. Choi K. C. Hwang K. A. (2018). Treatment with phytoestrogens reversed triclosan and bisphenol A-induced anti-apoptosis in breast cancer cells. Biomol. Ther. Seoul. 26 (5), 503–511. 10.4062/biomolther.2017.160 29310425 PMC6131008

[B22] Lee J. S. Han J. W. Jung M. Lee K. W. Chung M. S. (2020). Effects of thawing and frying methods on the formation of acrylamide and polycyclic aromatic hydrocarbons in chicken meat. Foods 9 (5), 573. 10.3390/foods9050573 32375322 PMC7278627

[B23] Li Z. Sandau C. D. Romanoff L. C. Caudill S. P. Sjodin A. Needham L. L. (2008). Concentration and profile of 22 urinary polycyclic aromatic hydrocarbon metabolites in the US population. Environ. Res. 107 (3), 320–331. 10.1016/j.envres.2008.01.013 18313659

[B24] Li L. X. Hao Y. Dong L. Qiao Z. Q. Yang S. C. Chen Y. D. (2025). Circular RNAs as biomarkers in breast cancer diagnosis, prognosis, molecular types, metastasis and drug resistance. Technol. Cancer Res. Treat. 24, 15330338251328500. 10.1177/15330338251328500 40080898 PMC11907621

[B25] Lian G. Y. Wang Q. M. Mak T. S. K. Huang X. R. Yu X. Q. Lan H. Y. (2021). Inhibition of tumor invasion and metastasis by targeting TGF-β-Smad-MMP2 pathway with Asiatic acid and naringenin. Mol. Ther. Oncolytics 20, 277–289. 10.1016/j.omto.2021.01.006 33614911 PMC7873580

[B26] Liu Y. Li X. R. Wang W. Yin B. Gao Y. Yang X. (2020). Chemical characteristics of atmospheric PM10 and PM2.5 at a rural site of lijiang city, China. Int. J. Environ. Res. Public Health 17 (24), 9553. 10.3390/ijerph17249553 33419360 PMC7765913

[B27] Liu G. Chen J. Bao Z. C. (2023). Promising antitumor effects of the curcumin analog DMC-BH on colorectal cancer cells. Aging-Us 15 (6), 2221–2236. 10.18632/aging.204610 PMC10085616 36971681

[B28] Lloyd V. Morse M. Purakal B. Parker J. Benard P. Crone M. (2019). Hormone-like effects of bisphenol A on p53 and estrogen receptor alpha in breast cancer cells. Biores Open Access 8 (1), 169–184. 10.1089/biores.2018.0048 31681507 PMC6823605

[B29] Mallah M. A. Li C. X. Mallah M. A. Noreen S. Liu Y. Saeed M. (2022). Polycyclic aromatic hydrocarbon and its effects on human health: an overeview. Chemosphere 296, 133948. 10.1016/j.chemosphere.2022.133948 35151703

[B30] Montano L. Baldini G. M. Piscopo M. Liguori G. Lombardi R. Ricciardi M. (2025). Polycyclic aromatic hydrocarbons (PAHs) in the environment: occupational exposure, health risks and fertility implications. Toxics 13 (3), 151. 10.3390/toxics13030151 40137477 PMC11946043

[B31] Mordukhovich I. Beyea J. Herring A. H. Hatch M. Stellman S. D. Teitelbaum S. L. (2016). Vehicular traffic-related polycyclic aromatic hydrocarbon exposure and breast cancer incidence: the long island breast cancer study project (LIBCSP). Environ. Health Perspect. 124 (1), 30–38. 10.1289/ehp.1307736 26008800 PMC4710589

[B32] Niebauer E. Fry N. Auster-Gussman L. A. Wahbeh H. (2021). Patient perspectives on the causes of breast cancer: a qualitative study on the relationship between stress, trauma, and breast cancer development. Int. J. Qual. Stud. Health Well-being 16 (1), 1983949. 10.1080/17482631.2021.1983949 34694978 PMC8547822

[B34] Peng C. X. Chen Y. N. Jiang M. Z. (2024). Targeting ferroptosis: a promising strategy to overcome drug resistance in breast cancer. Front. Oncol. 14, 1499125. 10.3389/fonc.2024.1499125 39759144 PMC11695291

[B35] Petrón M. J. Martín-Mateos M. J. Sánchez-Ordóñez M. Godoy B. Ramírez-Bernabé M. R. (2025). Antioxidant and quality effects of red grape pomace in barbecued pork burgers: implications for PAH formation. Antioxidants 14 (7), 832. 10.3390/antiox14070832 40722935 PMC12291812

[B36] Quoc T. T. Bácskay I. Fehér P. Pallér Á. Papp B. Bíró K. (2023). Personalized nasal protective devices: importance and perspectives. Life (Basel) 13 (11), 2116. 10.3390/life13112116 38004256 PMC10672262

[B37] Sato K. Osaka E. Fujiwara K. Fujii R. Takayama T. Tokuhashi Y. (2022). miRNA-218 targets multiple oncogenes and is a therapeutic target for osteosarcoma. Oncol. Rep. 47 (5), 92. 10.3892/or.2022.8303 35293593 PMC8968766

[B38] Saxena K. Jolly M. K. Balamurugan K. (2020). Hypoxia, partial EMT and collective migration: emerging culprits in metastasis. Transl. Oncol. 13 (11), 100845. 10.1016/j.tranon.2020.100845 32781367 PMC7419667

[B39] Shen J. Liao Y. Hopper J. L. Goldberg M. Santella R. M. Terry M. B. (2017). Dependence of cancer risk from environmental exposures on underlying genetic susceptibility: an illustration with polycyclic aromatic hydrocarbons and breast cancer. Br. J. Cancer 116 (9), 1229–1233. 10.1038/bjc.2017.81 28350789 PMC5418454

[B40] Song W. T. Liu Q. S. Sun Z. D. Yang X. Zhou Q. Jiang G. (2018). Polyfluorinated iodine alkanes regulated distinct breast cancer cell progression through binding with estrogen receptor alpha or beta isoforms. Environ. Pollut. 239, 300–307. 10.1016/j.envpol.2018.04.037 29665550

[B41] Steckley D. Karajgikar M. Dale L. B. Fuerth B. Swan P. Drummond-Main C. (2007). Puma is a dominant regulator of oxidative stress induced bax activation and neuronal apoptosis. J. Neurosci. 27 (47), 12989–12999. 10.1523/jneurosci.3400-07.2007 18032672 PMC6673275

[B42] Vérollet C. Charrière G. M. Labrousse A. Cougoule C. Le Cabec V. Maridonneau-Parini I. (2011). Extracellular proteolysis in macrophage migration: losing grip for a breakthrough. Eur. J. Immunol. 41 (10), 2805–2813. 10.1002/eji.201141538 21953638

[B43] Vita M. Henriksson M. (2006). The myc oncoprotein as a therapeutic target for human cancer. Seminars Cancer Biol. 16 (4), 318–330. 10.1016/j.semcancer.2006.07.015 16934487

[B44] Wang X. Y. Ren L. H. Zha W. G. Li Z. Dai R. Wang Z. (2022). Removal of p-toluenesulfonic acid from wastewater using a filtration-enhanced electro-Fenton reactor. Rsc Adv. 12 (39), 25424–25432. 10.1039/d2ra04921j 36199312 PMC9451130

[B45] Wen J. Y. Wang H. W. Dong T. J. Gan P. Fang H. Wu S. (2019). STAT3-induced upregulation of lncRNA ABHD11-AS1 promotes tumour progression in papillary thyroid carcinoma by regulating miR-1301-3p/STAT3 axis and PI3K/AKT signalling pathway. Cell. Prolif. 52 (2), e12569. 10.1111/cpr.12569 30657221 PMC6495520

[B46] Wu Z. S. Sun Z. H. Liu P. Y. Li Q. Yang R. Yang X. (2020). Competitive adsorption of naphthalene and phenanthrene on walnut shell based activated carbon and the verification *via* theoretical calculation. Rsc Adv. 10 (18), 10703–10714. 10.1039/c9ra09447d 35492953 PMC9050373

[B47] Yang Z. Cheng B. Song J. Wan Y. Wang Q. Cheng B. (2007). Estrogen accelerates G1 to S phase transition and induces a G2/M phase-predominant apoptosis in synthetic vascular smooth muscle cells. Int. J. Cardiol. 118 (3), 381–388. 10.1016/j.ijcard.2006.07.049 17055086

[B48] Zhang Q. Deng S. S. Li Q. C. Wang G. Guo Z. Zhu D. (2022). Glycoprotein M6A suppresses lung adenocarcinoma progression *via* inhibition of the PI3K/AKT pathway. J. Oncol. 2022, 4601501. 10.1155/2022/4601501 36405247 PMC9674424

[B49] Zhang P. Li S. L. Zeng H. Sun Y. (2025). Exposure to polycyclic aromatic hydrocarbons and bone mineral density in children and adolescents: results from the 2011-2016 national health and nutrition examination survey. Front. Public Health 13, 1428772. 10.3389/fpubh.2025.1428772 40313492 PMC12043670

[B50] Zhang X X. Wu W. Li X. He F. Zhang L. (2023). SPAG5 promotes the proliferation, migration, invasion, and epithelial-mesenchymal transformation of colorectal cancer cells by activating the PI3K/AKT signaling pathway. Chin. J. Physiol. 66 (5), 365–371. 10.4103/cjop.CJOP-D-22-00165 37929348

[B51] Zhang Y Y. Hu Q. Fu J. Li X. Mao H. Wang T. (2023). Influence of exposure pathways on tissue distribution and health impact of polycyclic aromatic hydrocarbon derivatives. Environ. Health (Wash). 1 (3), 150–167. 10.1021/envhealth.3c00060 39473616 PMC11503884

[B52] Zhou Z. Q. Sicairos B. Zhou J. H. Du Y. (2024). Proteomic analysis reveals major proteins and pathways that mediate the effect of 17-β-Estradiol in cell division and apoptosis in breast cancer MCF7 cells. J. Proteome Res. 23 (11), 4835–4848. 10.1021/acs.jproteome.4c00102 39392593 PMC11536429

